# Sarcomatoid renal cell carcinoma with an inferior vena cava tumor thrombus that was completely resected by robot-assisted laparoscopic radical nephrectomy after neoadjuvant therapy nivolumab plus ipilimumab: a case report

**DOI:** 10.1007/s13691-023-00627-5

**Published:** 2023-08-19

**Authors:** Ayaka Tsuchiyama, Kojiro Ohba, Hiromi Nakanishi, Takuji Yasuda, Yuichiro Nakamura, Hirokazu Kurohama, Kensuke Mitsunari, Tomohiro Matsuo, Yasushi Mochizuki, Ryoichi Imamura

**Affiliations:** 1 Department of Urology and Renal Transplantation, Nagasaki University Hospital, Sakamoto 1-7-1, Nagasaki, Japan; 2 Department of Pathology, Nagasaki University Hospital, Sakamoto 1-7-1, Nagasaki, Japan

**Keywords:** Inferior vena cava tumor thrombus, Ipilimumab, Nivolumab, Renal cell carcinoma, Robot-assisted laparoscopic radical nephrectomy

## Abstract

We here present a patient with a sarcomatoid renal cell carcinoma complicated by inferior vena cava tumor thrombus that we treated with nivolumab plus ipilimumab. This resulted in shrinkage of the tumor, enabling complete resection by robot-assisted laparoscopic radical nephrectomy. The patient is still alive with no evidence of recurrence.

## Introduction

Nivolumab plus ipilimumab was approved for treating chemotherapy-naive, curatively unresectable, and metastatic renal cell carcinoma (RCC) in Japan in August 2018. We here report a patient with sarcomatoid RCC with an inferior vena cava (IVC) tumor thrombus who was successfully treated with a combination of nivolumab plus ipilimumab as neoadjuvant therapy, followed by robot-assisted, laparoscopic, radical nephrectomy (RARN).

## Case report

**Fig. 1:**
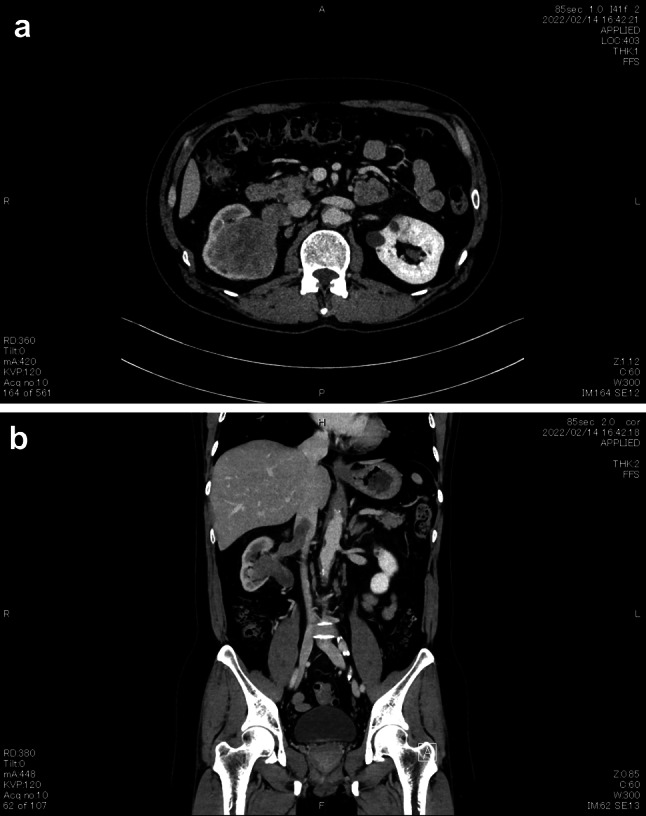
CT images of the right renal tumor on presentation. **a** CT image showing a 75- × 60-mm right renal tumor. **b** Contrast-enhanced CT image showing thrombus within the renal vein and a 35-mm-long thrombus in the inferior vena cava

A 58-year-old man was found to have impaired renal function. He also had hypertension, depression, and diverticulitis. His Karnofsky performance status was 100. Computed tomography (CT) revealed a right-sided renal tumor. Blood examination showed normal leukocyte and platelet counts and a normal hemoglobin concentration. C-reactive protein was 0.32 mg/dL, blood urea nitrogen was 16 mg/dL, serum creatinine was 1.27 mg/dL, estimated glomerular filtration rate was 46.57 mL/min/1.73m^2^, and corrected calcium concentration was 9.1 mg/dL. Urine examination showed no abnormalities. Contrast-enhanced CT revealed a 75- × 60-mm tumor in the right kidney. It extended from the renal parenchyma to the renal pelvis and was partially heterogeneous on contrast. A thrombus extending into the renal vein and inferior vena cava (IVC) to inferior to the liver was identified. The intra-IVC thrombus was 35 mm long (Mayo classification: Level 2) [1]. The maximum diameter of the venous tumor thrombus (VTT) was 17 mm and of the IVC 26 mm (Fig. 1). No metastases were identified.

**Fig. 2:**
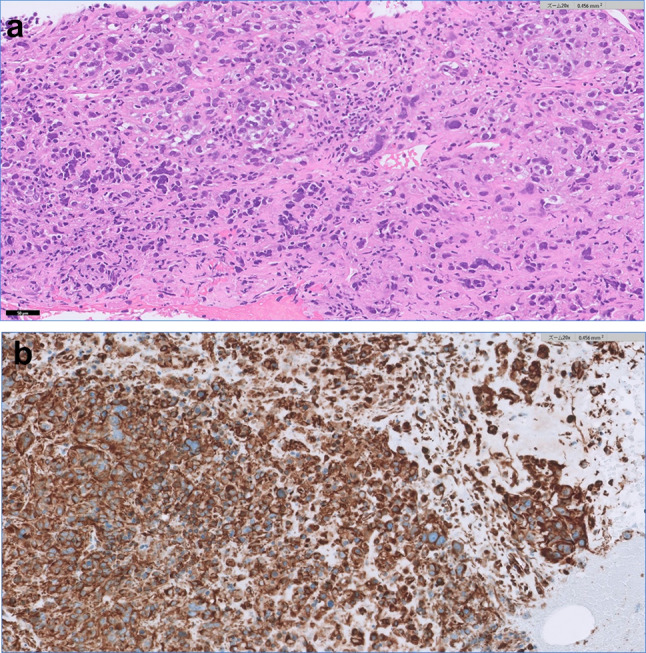
Photomicrographs of the biopsy specimen. **a** Hematoxylin–eosin-stained photomicrograph (20 ×). Polymorphic heterozygous cells are irregularly abundant and show spindle-shaped and sarcomatoid changes. **b** Vimentin-stained photomicrograph showing extensive positivity

**Fig. 3:**
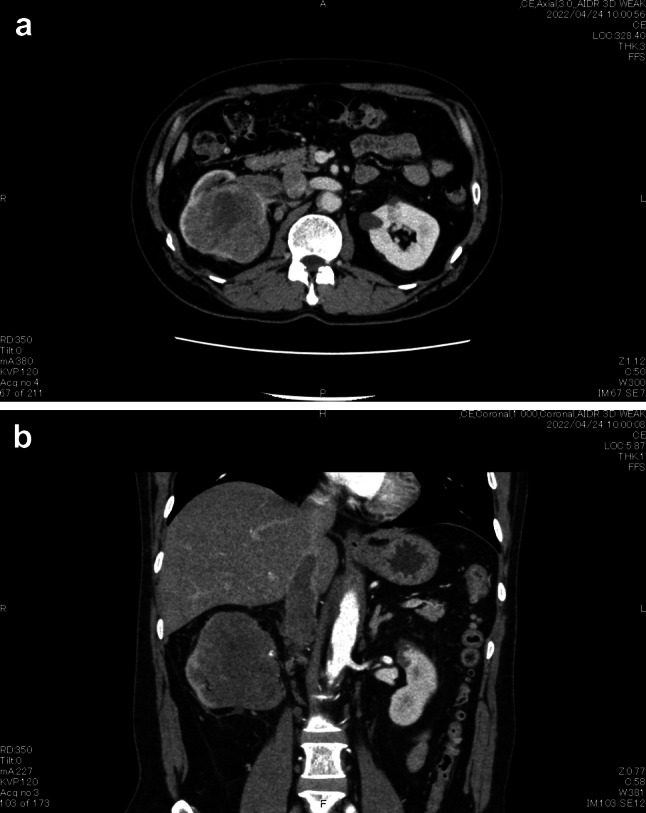
Images of the right renal tumor 3 months after presentation. **a** CT image showing a 78- × 76-mm right renal tumor. **b** Contrast-enhanced CT image showing a thrombus within the renal vein and a 77-mm-long thrombus in the inferior vena cava

**Fig. 4:**
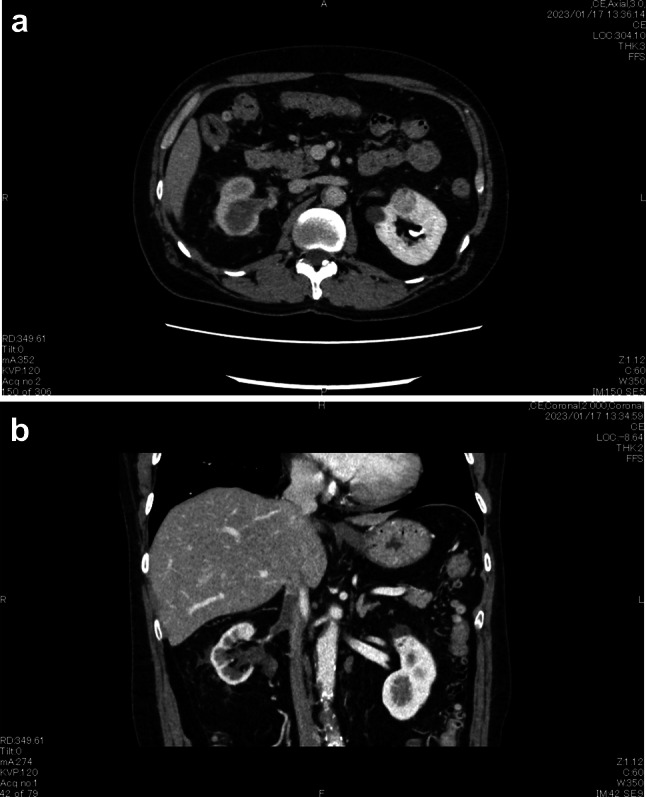
CT images of the right renal tumor after nivolumab plus ipilimumab (1 year after presentation). **a** The primary tumor has shrunk to 41 × 40 mm. **b** The maximum diameter of the thrombus is 8 mm and its length is 26 mm after the chemotherapy

Our provisional diagnosis was RCC, cT3bN0M0, stage III. However, we considered it necessary to differentiate it from a renal pelvic carcinoma. A CT-guided percutaneous tumor biopsy resulted in a pathological diagnosis of sarcomatoid RCC (Fig. 2). Surgery was scheduled for 1 month after biopsy, during which time the tumor grew rapidly. Immediately before planned surgery, the primary tumor was 78 × 76 mm, maximum diameter of the IVC-VTT 33 mm, and the intra-IVC-VTT 77 mm long, its head reaching the hepatic venous confluence (Fig. 3). Furthermore, his previously normal sized hilar lymph nodes had enlarged. It was considered that the tumor’s rapid progression made surgery too risky. According to the International Metastatic Renal Cell Carcinoma Database Consortium, his tumor was in the intermediate risk group. We, therefore, started treatment with nivolumab plus ipilimumab (nivolumab at 240 mg plus ipilimumab at 1 mg/kg intravenously every 3 week for four doses, followed by nivolumab at 480 mg every 4 weeks). The tumor shrank quickly but myositis, an immune-related adverse effect, developed after two cycles. The myositis resolved with prednisolone treatment, so nivolumab plus ipilimumab were completed for four doses, followed by three doses of nivolumab alone (480 mg). By 1 year after the initial presentation, the primary tumor had shrunk to 41 × 40 mm, the lymph nodes were of normal size, the maximum diameter of the thrombus was 8 mm, and the intra-IVC-VTT was only 26 mm long (Fig. 4). Considering that the tumor could then be removed surgically, we performed a RARN with IVC tumor thrombectomy (IVCTT). The right kidney was freed and a lymphadenectomy performed uneventfully. A rubber, double-loop, vascular band was used for a tourniquet and the distal end of the IVC, left renal vein, and proximal end of the IVC sequentially blocked with Hem-o-lock clips plus bulldog clamps. The anterior IVC wall was cut open. Although the IVC-VTT was slightly adherent to the vein wall, the entire tumor capsule was detached, extracted from the blood vessels, and completely removed. After which the opened IVC wall was closed with 5-0 polypropylene sutures and the IVC lumen was flushed with heparinized saline before being closed. Operative time was 384 min, console time was 287 min, and blood loss was 260 mL. There were no postoperative complications and the patient was discharged 7 days after surgery.

**Fig. 5:**
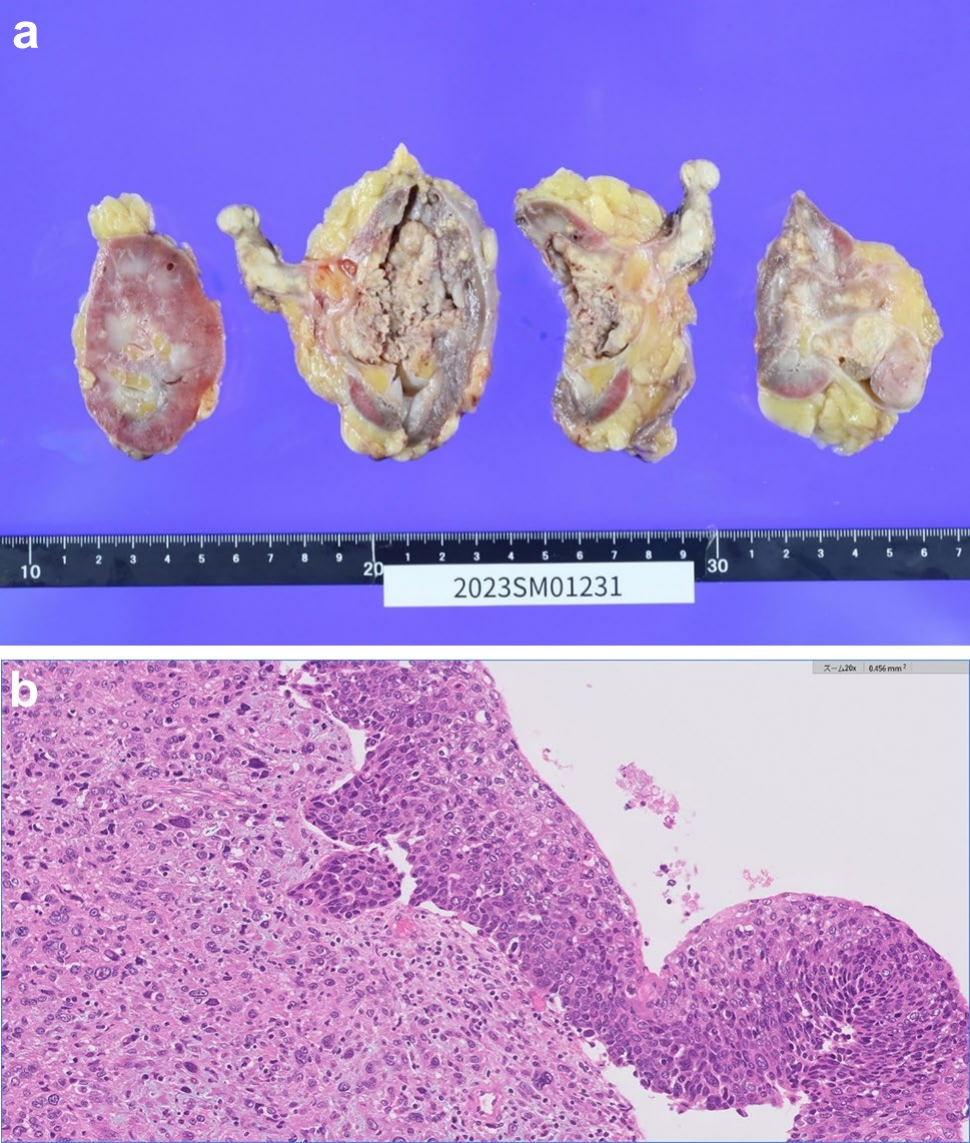
Images of resected tumor. **a** Photograph of the resected specimen (renal tumor and tumor thrombus). There is a large yellowish-white tumor in the kidney, 80% of which shows necrosis and fibrosis. **b** Hematoxylin–eosin-stained photomicrograph (20 ×) showing areas with epithelial connections and areas with sarcomatoid changes

Histological examination of the operative specimen showed a highly invasive sarcomatoid RCC extending into the renal pelvis and renal vein. Most of the tumor in the vein was necrotic. Additionally, 80% of the renal tumor was necrotic and fibrotic, indicating that the neoadjuvant immunotherapy had been effective. The resection margins were negative, and lymphocytic infiltration within the tumor is recognized in some tumor areas (Fig. 5). The final pathological diagnosis was sarcomatoid renal cell carcinoma, without other histologic types, pT3aN0M0, and WHO/ISUP Grade 4. At follow-up 3 months postoperatively, there was no evidence of recurrence.

## Discussion

The present patient had a T3b RCC without metastasis to other organs and was scheduled for surgery. However, his cancer progressed so rapidly that we considered that the risks of systemic spread and adverse perioperative events were unacceptably high and instituted neoadjuvant therapy. Having determined by biopsy that the tumor was an RCC and in light of published reports of the efficacy of a combination of nivolumab plus ipilimumab against these tumors, we chose this combination for our patient.

Nivolumab, an anti-programmed death receptor-1 antibody, exerts its antitumor effect by inhibiting binding between programmed death receptor-1 expressed on T cells and the corresponding ligands (PD-L1) expressed on tumors. Ipilimumab is a monoclonal antibody against cytotoxic T lymphocyte antigen-4 (CTLA-4), which is known to inhibit binding of CD80 and CD86 molecules on antigen-presenting cells, thereby causing proliferation and activation of T cells and promoting presentation of tumor antigen [2]. The phase III CheckMate 214 trial on patients with International Metastatic Renal Cell Carcinoma Database Consortium category intermediate-/poor-risk renal cell carcinoma showed that nivolumab plus ipilimumab achieved significantly longer overall survival and higher objective response rates than did sunitinib alone [3]. In addition, the combination of nivolumab plus ipilimumab reportedly achieves better outcomes in patients with strong PD-L1 expression. Furthermore, it has been reported that PD-L1 expression is strong in sarcomatoid RCC [4]. Survival benefit in patients with sarcomatoid RCC treated with nivolumab plus ipilimumab has recently been reported [5]. Other reports showed while exploratory analysis demonstrated similar response to immune checkpoint inhibitor plus vascular endothelial growth factor receptor—tyrosine kinase inhibitor in the sarcomatoid subgroup than in those without sarcomatoid RCC, post hoc analysis of CheckMate 214 demonstrated an improved response rate for combination of nivolumab plus ipilimumab in patients with sarcomatoid differentiation than in those without [6]. Furthermore, this patient had only tolerable symptoms and was not in urgent situation, and wanted a cure because of absent of distant metastasis. We selected nivolumab plus ipilimumab for our patient on the basis of these reports and the tumor size decreased markedly. Some studies have found that the combination of nivolumab plus ipilimumab can achieve regression of VTT [7–10]. It can, therefore, be expected that this combination may be so effective that it makes curative surgery for sarcomatoid RCC with IVC-VTT, which is otherwise difficult to operate on, possible. Additionally, the combination of nivolumab plus ipilimumab can reportedly achieve durable responses. However, there is a risk of immune-related adverse effects and no clear criteria for when to proceed with surgery. The optimal use of systemic treatment preceding resection of a primary tumor has not yet been adequately studied. We look forward to accumulation of cases and data from prospective studies.

There have been several reports of patients with metastatic RCC with IVC-VTT that shrank with the combination of nivolumab and ipilimumab, enabling cytoreductive nephrectomy [7–10]. There have also been two reported cases of complete resection of RCC with VTT without metastasis [11, 12]. However, to our knowledge, there have been no reported cases of successful combination therapy with nivolumab plus ipilimumab for sarcomatoid RCC with IVC-VTT. We, therefore, believe that this is the first reported case of RARN with level II IVCTT after neoadjuvant nivolumab plus ipilimumab. Nephrectomy requiring IVC-VTT often requires liver mobilization and the use of cardiopulmonary bypass, which increases the risk of perioperative complications and death [13]. Robot-assisted surgery enables reliable, shake-free procedures under a clear, magnified field of view with high image quality. It can, therefore, achieve less blood loss and reduce the risk of complications compared with conventional open surgery [14]. In the present case, robot-assisted surgery achieved little blood loss and the planned procedure was completed safely. We expect it will be more widely used in the future.

In conclusion, in our patient, a combination of nivolumab plus ipilimumab for sarcomatoid renal cell carcinoma with IVC-VTT resulted in tumor shrinkage. This enabled complete resection of the tumor by RARN with IVCTT, reducing surgical risks, such as blood loss and perioperative complications.
